# Tailored communication methods as key to implementation of evidence-based solutions in primary child health care

**DOI:** 10.1093/eurpub/ckaa234

**Published:** 2020-12-17

**Authors:** Kinga Zdunek, Peter Schröder-Bäck, Denise Alexander, Eline Vlasblom, Paul Kocken, Michael Rigby, Mitch Blair

**Affiliations:** 1 Department of Public Health, Medical University of Lublin, Lublin, Poland; 2 Department of International Health, Care and Public Health Research Institute (CAPHRI), Maastricht University, Maastricht, The Netherlands; 3 Section of Paediatrics, Imperial College London, London, UK; 4 School of Nursing and Midwifery, Trinity College Dublin, the University of Dublin, Dublin, Ireland; 5 Department of Child Health, TNO, Leiden, The Netherlands; 6 Erasmus School of Social and Behavioural Sciences, Erasmus University Rotterdam, Rotterdam, The Netherlands; 7 School of Social, Political and Global Studies and School of Primary, Community and Social Care, Keele University, Keele, UK; 8 Department of Primary Care and Public Health, Imperial College London, London, UK

## Abstract

**Background:**

Evidence-based policies should underpin successful implementation of innovations within child health care. The EU-funded Models of Child Health Appraised project enabled research into effective methods to communicate research evidence. The objective of this study was to identify and categorize methods to communicate evidence-based research recommendations and means to tailor this to stakeholder audiences.

**Methods:**

We conducted an online survey among national stakeholders in child health. Analysis of the most effective strategies to communicate research evidence and reach the target audience was carried out in order to ensure implementation of optimal child health care models at a national level.

**Results:**

Representatives of stakeholders from 21 of the then 30 EU MS and EEA countries responded to the questionnaire. Three main approaches in defining the strategies for effective communication of research recommendations were observed, namely: dissemination of information, involvement of stakeholders and active attitude towards change expressed in actions. The target audience for communicating recommendations was divided into two layers: proximal, which includes those who are remaining in close contact with the child, and distal, which contains those who are institutionally responsible for high quality of child health services. They should be recipients of evidence-based results communicated by different formats, such as scientific, administrative, popular and personal.

**Conclusions:**

Influential stakeholders impact the process of effective research dissemination and guide necessary actions to strengthen the process of effective communication of recommendations. Communication of evidence-based results should be targeted to each audience’s profile, both professional and non-professionals, by adjusting appropriate communication formats.

## Introduction

Child oriented health policies are universally important. Moreover, one of the priorities of the Universal Health Coverage strategy of the World Health Organization (WHO) is Primary Health Care, which includes actions on improvement of maternal, newborn, child and adolescent health.^1^ The European Commission recognizes the need to protect the wellbeing of children, e.g. in terms of medicinal products^2^ or the promotion of child rights.^3^ Member States are also adapting their national policies along these lines, though the approaches to child-focussed health policy vary between two patterns: ‘On the one hand the child-focussed policies are part of wider health care and policy context, on the other they are devoted to children as a stand-alone approach’.^4^ However, an elusive aspect of child health service improvement is how new knowledge is adopted by policymakers nationally.

Evidence-based policymaking has been defined as ‘a set of rules and institutional arrangements designed to encourage transparent and balanced use of evidence in public policymaking’,^5^ but literature shows ‘the limited extent to which professionals utilize or draw upon research findings to determine or guide their actions’.^6^ Also ‘a solid research infrastructure is facilitating but not sufficient for evidence use’.^7^ Policy developments and service improvements do not happen by accident—they have to be created, accepted by stakeholders, and implemented Examination of the policy cycle^8^ shows that tailored communication between researchers, policymakers, professionals and the general population is crucial in enabling transition from research evidence, to policy adoption, and then implementation and continuation.

Implementation of evidence into new policies is seldom a stand-alone activity—policymakers frequently and wisely look to see what has been done in similar neighbouring countries, and a previous Horizon 2020 project—the Research Inventory of Child Health Europe—specifically focussed on cataloguing such evidence in Europe.^9^ However, knowledge and effectiveness do not exist in a vacuum, and context is significant. Transferability based on context is therefore a key concept when planning to implement evidence-based policies found to work in one context in another country. The theory of transferability was developed in the Models of Child Health Appraised (MOCHA) study (as below), and comprises four key over-arching themes. In these themes, the population (P), the intervention (I) and the environment (E) represent a set of conditional transferability criteria, and the transfer of the intervention (T) represents process criteria for transferring the intervention to the target context, while overall transferability (−T) depends on the dynamic interaction.^10^

There is also a distinction to be made between top-down policy implementation by instruction, and enthusiastic adoption of the practices at the delivery level, and this depends on making the underpinning evidence accessible and credible. This requires effective knowledge communication relevant to specific recipients.

The goal of our research was to identify effective methods of communicating evidence to facilitate effective policy implementation, including identification of key audiences, drawing from practical experience in European Union (EU) and European Economic Area (EEA) countries.

## Methods

### Study design

This study was part of the EU-funded MOCHA project, which intended to assess various models of primary child health care across Europe,^11^ and had already identified three patterns: paediatrician-led, GP-lead or combined.^12^ In this inquiry, relevant stakeholders were identified, and a questionnaire was developed to measure the best possible ways to communicate evidence to appropriate recipients.

### Topic of inquiry

The types of stakeholder and relevant topics of inquiry were developed from consultation with fellow MOCHA researchers who were asked to identify key elements for the future of primary child health care. They focussed on domains, such as prevention, mental health, chronic care and complex care.^13^ This was then refined into specific activities in primary care reflecting these domains: (i) prevention of communicable diseases through vaccination of young children, (ii) treatment and monitoring of a chronic childhood condition and (iii) problem recognition/early diagnosis of mental health disorder in adolescents.

### Participants

The stakeholder selection process was achieved via the MOCHA Country Agents (CAs), who were national experts from the study countries who were recruited for the project in order to provide country-specific information. CAs were asked to identify, and supply contact details of, at least three stakeholders in their country who would be willing to complete a questionnaire about three broad areas of primary care, and three broad age groups of children as users of primary care. We highlighted that these stakeholders might be policymakers, physicians, school health doctors, paediatricians, nurses or others, but they needed to be knowledgeable about the healthcare system in the country. We asked them to include at least one policymaker in the field of primary child health care on a national level. In addition, European Union for School and University Health and Medicine (EUSUHM) congress members provided the names of relevant national stakeholders in their countries. The stakeholders were asked to respond to the questionnaire based on their expert knowledge and experience and expertise, not their personal opinions.

### Questionnaire

Stakeholders were asked to complete a digital questionnaire about communication modes to ensure implementation of evidence-based solutions in their countries:

- the most effective strategy for communicating recommendations, to ensure implementation of optimal models,
- the most effective target audience for promoting implementation of optimal models and
- the most effective format for communicating policy evidence.

In line with the MOCHA project’s established methodology, the questionnaire was designed by the topic researchers, approved by the project coordination team and validated by the project’s External Advisory Board, comprising members nominated by European medical, paediatric and policy bodies, WHO European Regional Office, UNICEF Innocenti Research Centre and civil society groups, as published.^14^ This ensured scientific and professional validity.

### Data collection

The data collection was carried out between March and May 2018. Out of the 30 EU/EEA countries, the MOCHA CAs and EUSUHM congress members of 22 countries provided names of 161 stakeholders.

### Data analysis

Our questions were open ended, and were analyzed by using thematic content analysis. The collected responses were coded by highlighting relevant parts of the answers. This facilitated further categorization, which led to emergence of umbrella themes characterizing strategies to implement evidence-based research recommendations and means to tailor this to the audiences. In order to identify, analyze and report patterns (themes) within the data, the approach proposed by Brown and Clarke^15^ with six phases was used.

The analytical process led to identification of clusters of strategies of effective communication of evidence-based data, format of recommendations and target audiences on which the stakeholders participating in the survey showed a convergence.

### Ethics

The study was reviewed and approved by the ethical committee of the Faculty of Behavioural, Management and Social Sciences of the University of Twente under file number BCE17614, on 19 September 2017.

## Results

**Figure 1:**
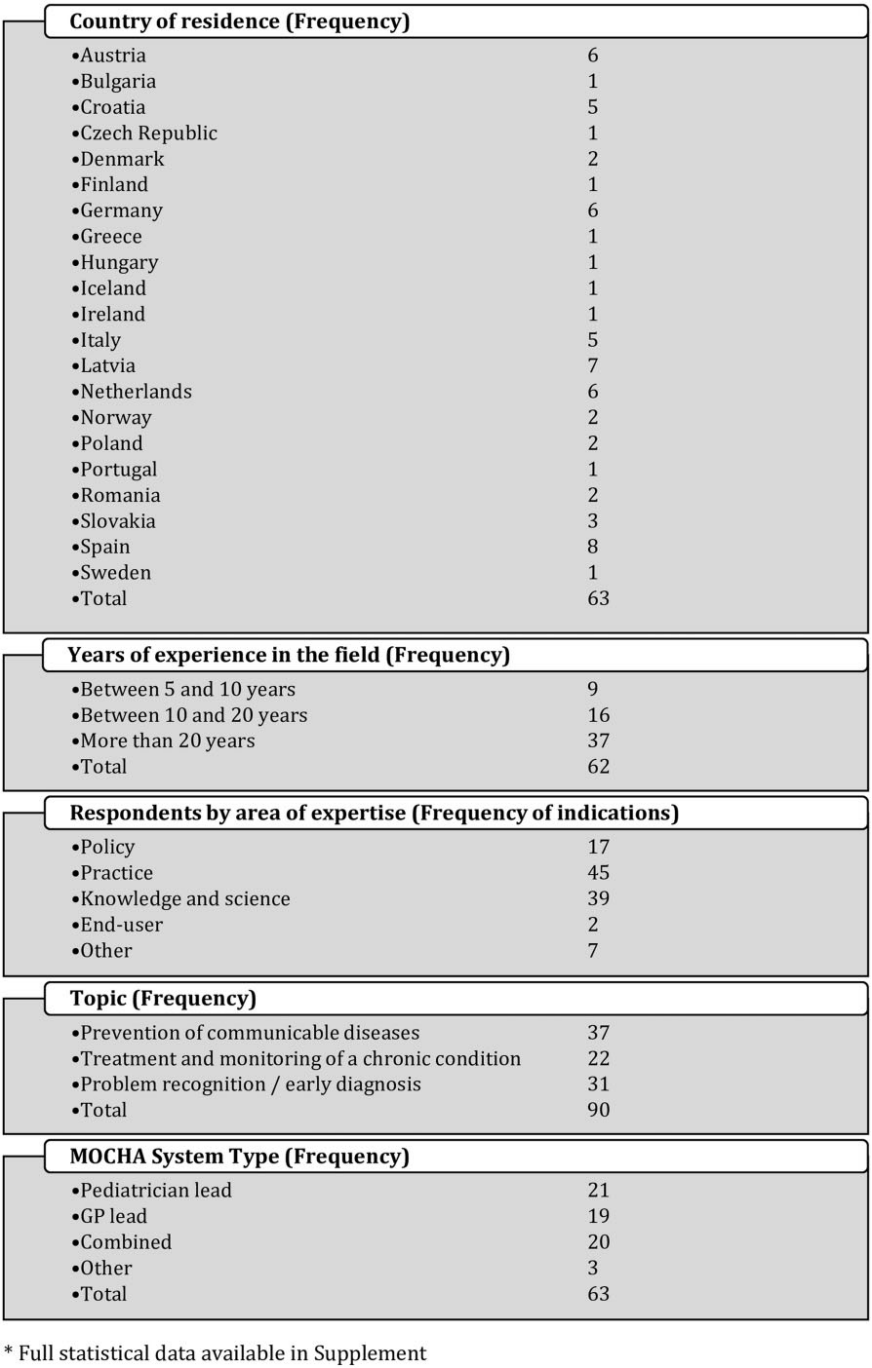
Characteristic of respondents

In total, 99 (61.5%) of 161 nominated stakeholders started the questionnaire, 90 (55.9%) completed it—they were from 21 countries comprising all EU Member States except Belgium, Cyprus, Estonia, France, Lithuania, Luxembourg, Malta, Slovenia and the UK, plus Norway and Iceland from the EEA (figure 1).

Most respondents were experts in prevention of communicable diseases (vaccination as a tracer), and recognition of mental health problems in adolescents. The least numerous group was experts in treatment and monitoring of a chronic condition (figure 1).

A total of 62 out of 90 respondents answered the questions about most effective strategy, target audience and format for communicating policy recommendations. They represented three types of MOCHA Primary Health Care system as identified by the MOCHA project: GP-lead (31.2%), Paediatrician-lead (33.3%) and Combined (31.7%) along with others (4.8%) (figure 1). Some of the stakeholders declared an expertise in more than one field: within the topic of treatment and monitoring of chronic condition (19 responses), prevention of communicable diseases (28 responses) and problem recognition and early diagnosis of mental health problems in adolescents (19 responses). Thus, we obtained 66 responses to the questions about evidence-based issues. Full statistical characteristic of respondents is available in the Supplementary tables S1–S5.

### Strategies for effective communication of recommendations

**Figure 2:**
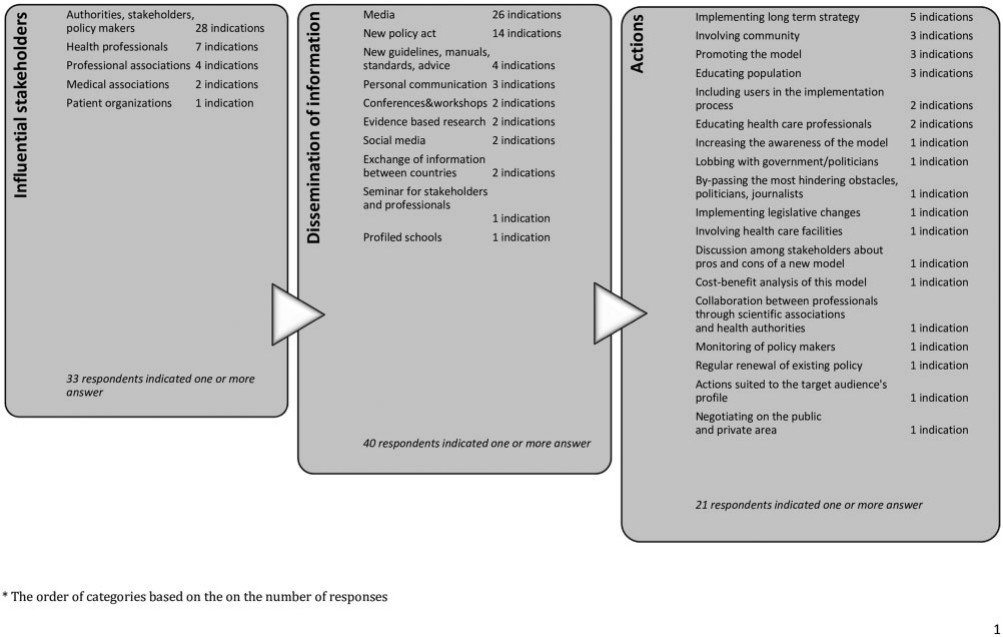
Approaches in defining the strategies of effective communication of recommendations

We identified three over-arching approaches to effective communication of policies (figure 2):

- influential stakeholders’ impact on communication processes regarding evidence-based recommendations for policies (36.2% responses),
- dissemination of information in order to provide effective communication of evidence-based recommendations for policies (42.6% responses) and
- necessary actions in order to strengthen the process of effective communication of evidence-based recommendations for policies (21.3% responses).

Consistently, dissemination played the most important role in each of the three groups of countries classified by system type (Supplementary table S6).

#### Influential stakeholders

The most influential stakeholders in disseminating the recommendations influencing optimal models of child health were authorities and policymakers, as those who are also responsible for further adoption and implementation of innovations. The significant role of health professionals and other associations (professional, medical and patient) was indicated as well (Supplementary table S7). The German respondent highlighted that ‘community/public and associations should be also convinced of the new idea in order to put pressure on politicians who would make the decisions’ (respondent 14, Germany).

#### Dissemination of information

Strategic tools for dissemination of information were identified regarding ‘hard’ law (legally binding) and ‘soft’ recommendations (not legally binding).^16^ Respondents mentioned that not only new formal policies, but also soft guidelines and recommendations were important. Seminars, conferences and workshops are significant facilitators of exchange of information, not only between countries but also between competent authorities. Experts highlighted the strategic role of media, including social media, in the dissemination of information about innovative solutions facilitating the process of active implementation (Supplementary table S8).

A Spanish respondent claimed that ‘legislation, policies, standards, advice and guidance are necessary to provide the framework for addressing critical issues such as the provision of care of high quality, the improvement of access to care, the protection of rights’ (respondent 49, Spain). It was stressed that strategies should be ‘suited to the target audience's profile’ (respondent 19), while media often determine what is visible for the public and politicians (respondent 22, Norway). The media has power and can lead to the mobilization of societal action that creates the conditions and place for health issues on the national public agenda and can catalyse action at the national and local levels (respondent 49, Spain).

#### Actions

In the respondents’ opinion actions should be based on the implementation of long-term strategies or legislative changes, with the involvement of users at professional and non-professional level. Promoting the model by spreading a positive message is key in the process of increasing awareness (Supplementary table S9).

The regular renewal of the existing action plan and program of health care measures was said to be important (respondent 69, Croatia). In order to obtain the broad scope of the recipients who are aware of new child healthcare evidence recommendations, discussion among stakeholders about pros and cons of a new model and cost-benefit analysis of this model is recommended (respondent 72, Latvia). Importance was also given to the meeting and personal encounters with authorities directly responsible for child health services (respondent 74, Sweden). The optimal strategy should be ‘through well planned, sufficiently funded implementation work that targets service providers directly with content that appears to be useful in their everyday work’ (respondent 7, Norway), emphasizing the targeting to the particular needs of each stakeholder in their work context. The Austrian expert in problem recognition and early diagnosis proposed a strategy, which was education based, with activities oriented to those who are working with the child in the field, to children and to parents (respondent 58, Austria).

### Target audience

In order to identify the most appropriate recipients who should be informed about the development of a new model, we asked respondents to identify the most effective target audience for communicating recommendations, to ensure successful implementation of optimal models in their countries. Experts recognized the significant importance of both patients and their environment at micro level as well as decision/policymakers, and professional associations and organizations at macro level. Many of them stressed that both the format of the recommendations, as well as the strategy, should be suited to the target audience.

Observing the data, we divided the reported target audience for communicating recommendations into two layers:

- audiences in the proximal environment of the child/patient (42.2% responses) and
- audiences in the distal environment of the child/patient (57.8% responses).

We noticed that experts from all three groups of MOCHA systems were choosing the distal audience as most relevant (Supplementary table S10).

#### Proximal audience

The proximal target audience consists of children/patients, families, parents, people supporting parents, self-help groups, health care workers, teachers and health professionals (Supplementary table S11). This group includes those who come into direct contact with the child and are the recipients of the implemented policies. In the opinion of respondents they should also be included in the group of recipients of evidence-based solutions as they can indirectly affect the policymaking process.

The importance of health professionals who have the power to change the system was stressed (respondent 22, Norway). The Spanish respondent highlighted that parents should be informed by primary care professionals about new evidence-based solutions (respondent 24, Spain).

#### Distal audience

The audience of the distal environment of the child includes: decision makers, professional organizations and associations, politicians, child advocacy groups, patient associations, administration—civil servants, health insurances, governmental institutions, opinion leaders, authorities (including local authorities), knowledge centres, general public/service users, journalists and health mediators (Supplementary table S12). This group includes specialists and decision makers who are directly involved in the policymaking process. The diversity of stakeholders mentioned by experts shows the need for adjusting the type of evidence to the various groups of recipients.

The Latvian stakeholder stressed that ‘politicians in particular in municipalities do not have a high level of health literacy, so they are definitely one target audience’ (respondent 32, Latvia). Also, governmental institutions should be included in the group of primary target audiences (respondent 30, Finland). Policymakers and other stakeholders need to have the expertise to examine state-level data and differentiate specific risk sub-populations (respondent 49, Spain).

### Format of the recommendations

**Figure 3:**
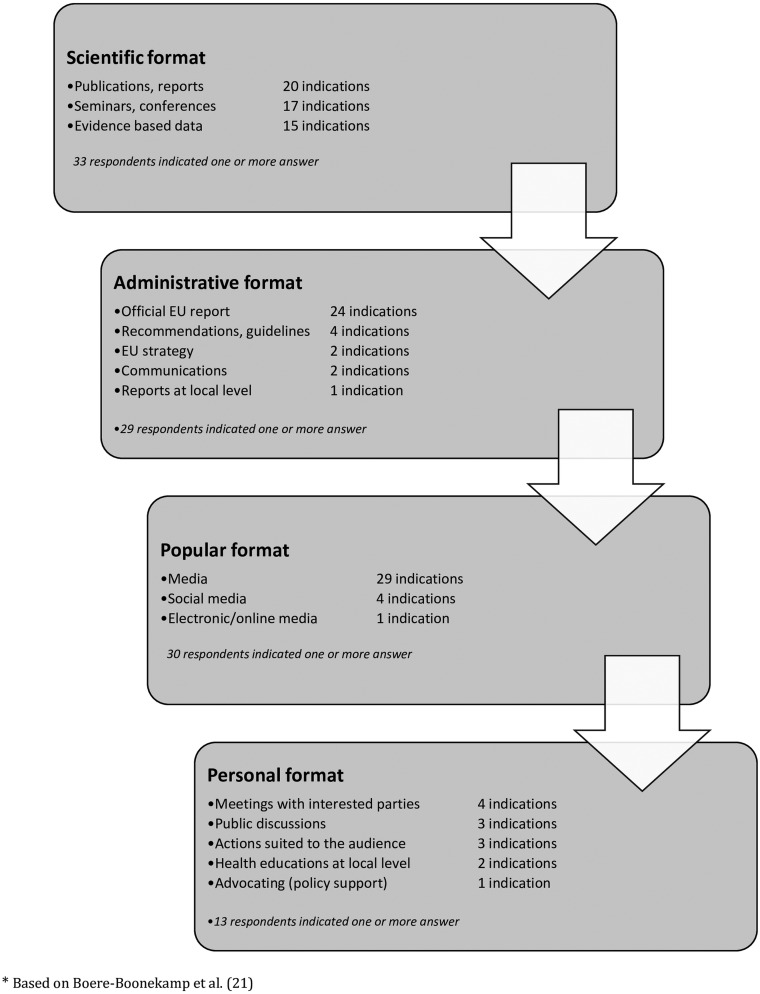
Types of most effective format of communication

Based on the data collected, we could observe that there are four types of effective formats of communication (figure 3):

- scientific format (33.1% responses),
- administrative format (27.4% responses),
- popular format (28.3% responses),
- personal format (13.2% responses).

On the one side, the scientific approach is still relevant, popular and expected (Supplementary table S13), together with the administrative and formalized reports, strategies and recommendations (Supplementary table S14). On the other hand, we noted that the data must be adapted to the general population and users who are more aware of the emerging possibilities of improvement of the quality of care and services. Thus, the popular format, which contains media, social media and electronic media were identified (Supplementary table S15). Additionally, there is the need for public involvement in the discussion of newly proposed solutions, which is correlated with health education activities at the primary care level/health personnel, meetings with parents and citizens, decision makers/citizens involvement, public discussions including competent authorities and/or celebrities (Supplementary table S16).

We also observed that the countries that are representing the combined and paediatrician-lead MOCHA system were mostly choosing the administrative format of recommendation as most relevant whereas GP-lead countries were preferring the scientific format. However, the differences were minimal (Supplementary table S17).

#### Scientific and administrative format

The answers given by the respondents confirm that the format of advice ‘should be suited to the target audience's profile, either individual or priority groups, i.e. peer-reviewed journal and/or seminar for stakeholders and professionals’ (respondent 19). The Norwegian expert highlighted that ‘reports, scientific publications, seminars and news items are either useless or make a temporary change. The format must appear useful for the person receiving it, and it must be followed up regularly to ensure actual implementation’ (respondent 7, Norway). A Croatian respondent stressed that ‘health professionals will like a peer-reviewed journal, politicians and decision makers would prefer EU report, and parents will react to the popular media’ (respondent 69, Croatia).

#### Popular and personal format

The most relevant and effective format for patients is media because ‘patients should know what is possible and might be better’ (respondent 44, Austria). Public discussions with doctors should be facilitated by famous persons/celebrities (particularly in terms of immunization), supported by educational shots in media, whereas scientific publications should be directed to medical professionals (respondent 66, Slovakia). Reports published in mass media and social networks, as well as innovative approaches, technology-based, and peer-led approaches, may increase awareness amongst patients and also general population (respondent 49, Spain).

A Latvian expert stressed that ‘evidence-based scientific publication in a peer-reviewed journal is good for scientists and writers but not for wider society’. He suggested that permanent and positive information in popular media and advocacy from the authorities could have the greatest benefit (respondent 32, Latvia). Others claimed that strong scientific evidence should be disseminated by social media (respondent 12, Italy).

## Discussion

**Figure 4:**
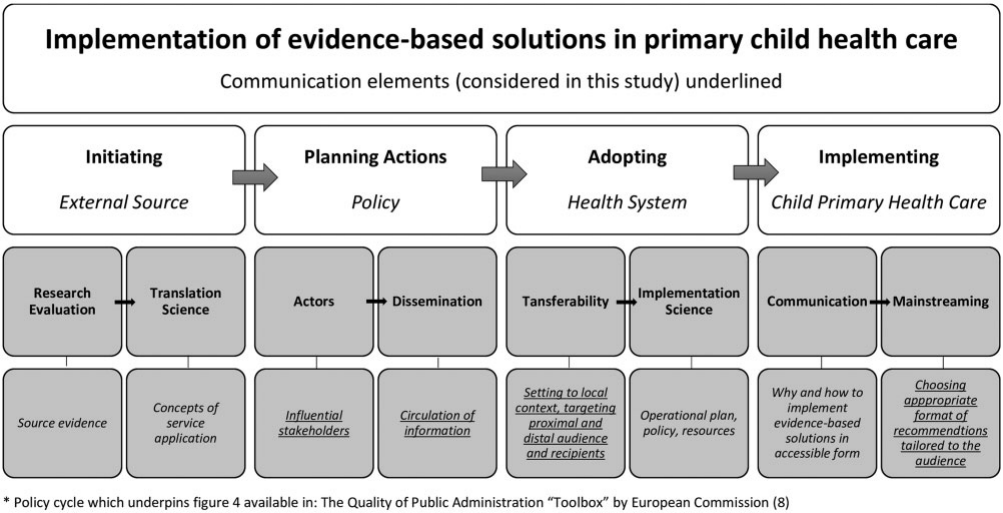
Implementation of evidence-based solutions in primary child health care

It is important to recognize that evidence-based policy, in order to be effective, needs to rely on appropriate strategies of dissemination of scientific results. Based on the analysis of stakeholders’ views, we characterized the strategies to communicate evidence-based research recommendations and means to tailor this to the audiences and we set it in the wider context of the recognized policy cycle^8^ (figure 4).

In our study, we identified three essential aspects that need to be taken into account while planning introduction of evidence-based innovation (model).

Firstly, stakeholders representing medical and patient environment should be considered as crucial component in the process of the dissemination of information. It is compatible with a definition of a stakeholder as ‘any group or individual who can affect or is affected by the developed (…) system’.^17^^,^^18^

Disseminated information about innovative solutions can take various forms and go through various channels. In particular media impact was highlighted, and that is consistent with the opinion of Van de Goor et al.^7^ that ‘media attitude towards underpinning policy with evidence influences policy decision makers’.

These findings support communicating evidence in a way that appeals to specific complementary audiences. They make the difference between mechanical acceptance of a policy and enthusiastic adoption and successful implementation. Effective dissemination requires active circulation of the evidence/research and leads to positive local innovation. It fits the view of Greenhalgh et al.^19^ that dissemination is an active, planned effort to persuade target groups to adopt an innovation, while implementation is an active and planned effort to mainstream it within an organization.^19^ The consequence of appropriate dissemination and actions is adoption, which is the series of stages from first hearing about a product to finally applying it.^19^

Successful transfer of the innovation requires tailoring the message to appropriate audiences. We identified two layers of the target audience; the proximal audience include those who are remaining in close contact with the child and who indirectly influence the policymaking processes, and the distal audience of those who are institutionally responsible child health services or play an advocacy role. This division is compatible with a classification identified by the MOCHA project where two groups of children’s agents were identified, agents of proximal and distal child environment.^20^

Eventually, this study presented a cascade view of the most effective format of recommendations (figure 3 based on Boere-Boonekamp et al.^21^), which shows the baseline for the process of communicating and mainstreaming evidence-based policy, including publications or other academic reports. Even though scientific data are the main source of the administrative recommendations and strategies ‘solid research infrastructure is facilitating but not sufficient for evidence use’.^7^ A powerful role is played by various kinds of media, which are significant channels for popularizing the research amongst a wider public. Messages and evidence that appear in popular media help to reach the recipient at a personal level.

To conclude, Influential stakeholders impact the process of effective research dissemination and guide necessary actions to strengthen the process of effective communication of recommendations. Communication of evidence-based results should be targeted to each audience’s profile, both professional and non-professionals, by adjusting appropriate communication formats.

### Strengths and limitations

Our work drew on respondents from a large number of diverse European countries who are active in the functions of primary health care and working with different age groups. However, it was not possible to include stakeholders from all European countries and of all fields in the research. We are aware that the 61% response rate might bring the risk of limited representativeness, but collecting the data from high level decision makers is challenging. However, they have been carefully chosen by CAs with criteria such as knowledgeable and national view.

We are aware that proposed recommendations may have very different relevance for different interventions and the choice of dissemination strategies. In applying the approaches emerging from the study at local level, national experts should adapt several approaches of communicating evidence, taking into account contextual determinants of child health policy, which we characterized in previous works.^22^

## Supplementary data

Supplementary data are available at *EURPUB* online.

## Funding

The article is part of the work of WP9 within the project MOCHA (Models of Child Health Appraised) that is funded by the European Commission through the Horizon 2020 Framework (grant agreement number: 634201).

## Conflicts of interest

None declared.

## Data availability

The MOCHA data contains no patient information, but may contain other personal or institutional data, such as source of a commentary. The MOCHA project has therefore resolved that source data will be curated on the MOCHA web site, and be accessible via the Principal or other Partners through a curator function, so that data relevant to any enquiry can be supplied, and redaction effected, but also contextualization given.

**Key points**

- There are three main approaches in defining the strategies of effective communication of recommendations, namely: dissemination of information, stakeholder impact and actions.
- Stakeholders are communicating the information in order to undertake significant national actions.
- The format of recommendations, as well as the strategy, should be tailored to the target audience.
- The audience of proximal environment includes those who are remaining in close (personal) contact with the child whereas the audience of distal environment contains those who are institutionally responsible for high quality of child health services in their countries.
- The baseline for the process of circulating the information is the evidence-based data of a scientific character as it is the main source of the administrative recommendations and reports, which later appear in broadly understood media, the channel, which reaches the recipient at personal level.

## Supplementary Material

ckaa234_Supplementary_Data
