# Supplementary material for: Tailored communication methods as key to implementation of evidence-based solutions in primary child health care
Source: Eur J Public Health. 2020 Dec 17;31(1):92–9. doi: 10.1093/eurpub/ckaa234 (PMC7851896; doi:10.1093/eurpub/ckaa234)
Supplement: ckaa234_Supplementary_Data [file ckaa234_supplementary_data.pdf]

Supplementary material

Analysis completed using – SPSS software

Table 1 Country of residence

Country of residence				
		Frequency	Percent	Valid Percent
Valid	Austria	6	6,1	9,5
	Bulgaria	1	1,0	1,6
	Croatia	5	5,1	7,9
	Czech Republic	1	1,0	1,6
	Denmark	2	2,0	3,2
	Finland	1	1,0	1,6
	Germany	6	6,1	9,5
	Greece	1	1,0	1,6
	Hungary	1	1,0	1,6
	Iceland	1	1,0	1,6
	Ireland	1	1,0	1,6
	Italy	5	5,1	7,9
	Latvia	7	7,1	11,1
	Netherlands	6	6,1	9,5
	Norway	2	2,0	3,2
	Poland	2	2,0	3,2
	Portugal	1	1,0	1,6
	Romania	2	2,0	3,2
	Slovakia	3	3,0	4,8
	Spain	8	8,1	12,7
	Sweden	1	1,0	1,6
	Total	63	53,6	100,0
Missing	8888888	29	29,3	
	9999999	7	7,1	
	Total	36	36,4	
Total		99	100,0	

Table 2 Topic of expertise

Topic of expertise				
		Frequency	Percent	Valid Percent
Valid	Prevention of communicable diseases (vaccination as a tracer for preventive care services)	37	37,4	41,1
	Treatment and monitoring of a chronic condition (optimising chronic health care e.g. asthma	22	22,2	24,4
	Problem recognition / early diagnosis (Early identification of mental health disorder) (adolescents)	31	31,3	34,4
	Total	90	90,9	100,0
Missing	8888888	9	9,1	
Total		99	100,0	

Table 3 Years of experience of respondents

**Years of experience in the field**				
		Frequency	Percent	Valid Percent
Valid	Between 5 and 10 years	9	9,1	14,5
	Between 10 and 20 years	16	16,2	25,8
	More than 20 years	37	37,4	59,7
	Total	62	62,6	100,0
Missing	8888888	29	29,3	
	9999999	8	8,1	
	Total	37	37,4	
Total		99	100,0	

Table 4 Area of expertise

Area of expertise				
		Responses		
		N	Percent	Percent of Cases
Area of expertise[a]	Policy	17	15,5%	26,6%
	Practice	45	40,9%	70,3%
	Knowledge and science	39	35,5%	60,9%
	End user (for example, representative of a patient advocacy group)	2	1,8%	3,1%
	Other	7	6,4%	10,9%
Total		110	100,0%	171,9%

64 respondents gave one or more answer

Table 5 MOCHA system type

MOCHA system type				
		Frequency	Percent	Valid Percent
Valid	Combined	20	20,2	31,7
	GP-lead	19	19,2	30,2
	Pediatrician-lead	21	21,2	33,3
	Other	3	3,0	4,8
	Total	63	63,6	100,0
Missing	System	36	36,4	
Total		99	100,0	

Table 6 Strategies for effective communication of recommendations by MOCHA System type

Strategies for effective communication of recommendations *MOCHA System type							
			MOCHA system type				
			Combined	GP-lead	Pediatrician-lead	Other	Total
Strategies for effective communication of recommendations [a]	Stakeholders impact	Count	10	9	12	2	33
		% within system type	34,5%	34,6%	38,7%	40,0%	
	Dissemination of information	Count	14	10	13	2	39
		% within system type	48,3%	38,5%	41,9%	40,0%	
	Actions	Count	5	7	6	1	19
		% within system type	17,2%	26,9%	19,4%	20,0%	
Total		Count	29	26	31	5	91
Percentages and totals are based on responses.							
a. Group							

Table 7 Influential stakeholders

Influential stakeholders				
		Responses		
		N	Percent	Percent of Cases
Influential stakeholders[a]	Authorities/stakeholders/policy makers	28	66,7%	84,8%
	Health professionals	7	16,7%	21,2%
	Professional associations	4	9,5%	12,1%
	Medical associations	2	4,8%	6,1%
	Patient organizations	1	2,4%	3,0%
Total		42	100,0%	127,3%
a. Dichotomy group tabulated at value 1.				

33 respondents gave one or more answers

Table 8 Dissemination of information

Dissemination of information				
		Responses		
		N	Percent	Percent of Cases
Dissemination of information[a]	Media	26	45,6%	65,0%
	New policy act	14	24,6%	35,0%
	New guidelines, manuals, standards, advice	4	7,0%	10,0%
	Personal communication	3	5,3%	7,5%
	Conferences and workshops	2	3,5%	5,0%
	Evidence based research (incl. peer-reviewed journal)	2	3,5%	5,0%
	Social media (incl. messenger based information)	2	3,5%	5,0%
	Exchange of information between countries	2	3,5%	5,0%
	Seminar for stakeholders/professionals	1	1,8%	2,5%
	Profiled schools	1	1,8%	2,5%
Total		57	100,0%	142,5%

a. Dichotomy group tabulated at value 1.

40 respondents gave one or more answers

Table 9 Actions

Actions				
		Responses		
		N	Percent	Percent of Cases
Actions[a]	Implementing long term strategy	5	16,7%	23,8%
	Involving community	3	10,0%	14,3%
	Promoting the model	3	10,0%	14,3%
	Educating population	3	10,0%	14,3%
	Including users in the implementation process	2	6,7%	9,5%
	Educating health care professionals	2	6,7%	9,5%
	Increasing the awareness of the model	1	3,3%	4,8%
	Lobbing with government/politicians	1	3,3%	4,8%
	By-passing the most hindering obstacles/politicians/journalists	1	3,3%	4,8%
	Implementing legislative changes	1	3,3%	4,8%
	Involving health care facilities	1	3,3%	4,8%
	Discussion among stakeholders about pros and cons of a new model	1	3,3%	4,8%
	Cost-benefit analysis of this model	1	3,3%	4,8%
	Collaboration between professionals through scientific associations and health authorities	1	3,3%	4,8%
	Monitoring of policy makers	1	3,3%	4,8%
	Regular renewal of existing policy	1	3,3%	4,8%
	Actions suited to the target audience's profile	1	3,3%	4,8%
	Negotiating on the public and private area	1	3,3%	4,8%
Total		30	100,0%	142,9%
a. Dichotomy group tabulated at value 1.				

21 respondents gave one or more answers

Table 10 Audience by MOCHA system type

Audience*MOCHA system type							
			MOCHA system type				Total
			Combined	GP-lead	Pediatrician-lead	Other	
Audience[a]	Proximal Audience	Count	14	11	9	3	37
		% within system type	43,8%	40,7%	37,5%	60,0%	
	Distal Audience	Count	18	16	15	2	51
		% within system type	56,3%	59,3%	62,5%	40,0%	
Total		Count	32	27	24	5	88
Percentages and totals are based on responses.							
a. Group							

Table 11 Proximal agents

Proximal agents				
		Responses		
		N	Percent	Percent of Cases
Proximal agents[a]	Health professionals	37	38,1%	69,8%
	Parents	28	28,9%	52,8%
	Children/Patients	20	20,6%	37,7%
	Teachers	4	4,1%	7,5%
	Health care workers	3	3,1%	5,7%
	Families	2	2,1%	3,8%
	People supporting parents/working with children	2	2,1%	3,8%
	Self-help groups	1	1,0%	1,9%
Total		97	100,0%	183,0%
a. Dichotomy group tabulated at value 1.				

53 respondents gave one or more answers

Table 12 Distal agents

Distal agents				
		Responses		
		N	Percent	Percent of Cases
Distal agents[a]	Decision makers	27	40,9%	69,2%
	Politicians	7	10,6%	17,9%
	Professional organizations/associations	6	9,1%	15,4%
	Stakeholders	5	7,6%	12,8%
	General public/Service users	4	6,1%	10,3%
	Health insurances	3	4,5%	7,7%
	Authorities inc. local	3	4,5%	7,7%
	Knowledge centres/scientific associations	3	4,5%	7,7%
	Patient associations	2	3,0%	5,1%
	Governmental institutions	2	3,0%	5,1%
	Administrators – civil servants	1	1,5%	2,6%
	Opinion leaders	1	1,5%	2,6%
	Journalists	1	1,5%	2,6%
	Health mediators	1	1,5%	2,6%
Total		66	100,0%	169,2%
a. Dichotomy group tabulated at value 1.				

39 respondents gave one or more answers

Table 13 Scientific format

**Scientific format**				
		Responses		
		N	Percent	Percent of Cases
Scientific format[a]	Publications/Reports	20	38,5%	60,6%
	Seminars/Conferences	17	32,7%	51,5%
	Evidence-based data	15	28,8%	45,5%
Total		52	100,0%	157,6%
a. Dichotomy group tabulated at value 1.				

33 respondents gave one or more answers

Table 14 Administrative format

Administrative format				
		Responses		Percent of Cases
		N	Percent	
Administrative format[a]	Official EU report	24	72,7%	82,8%
	Recommendations/Guidelines	4	12,1%	13,8%
	EU strategy	2	6,1%	6,9%
	Communications	2	6,1%	6,9%
	Reports at local level	1	3,0%	3,4%
Total		33	100,0%	113,8%
a. Dichotomy group tabulated at value 1.				

29 respondents gave one or more answers

Table 15 Popular format

Popular format				
		Responses		Percent of Cases
		N	Percent	
Popular format[a]	Media	29	85,3%	96,7%
	Social media	4	11,8%	13,3%
	Electronic media	1	2,9%	3,3%
Total		34	100,0%	113,3%

a. Dichotomy group tabulated at value 1.

30 respondents gave one or more answers

Table 16 Personal format

Personal format				
		Responses		
		N	Percent	Percent of Cases
Personal format[a]	Meetings with parents/ citizens/ decision makers/citizens involvement	4	30,8%	30,8%
	Public discussions (incl. competent authorities and/or celebrities)	3	23,1%	23,1%
	Actions suited to the target audience	3	23,1%	23,1%
	Health educations activities at the primary care level/health personnel level	2	15,4%	15,4%
	Advocating as a support for particular policy	1	7,7%	7,7%
Total		13	100,0%	100,0%

a. Dichotomy group tabulated at value 1.

13 respondents gave one or more answers

Table 12 Format of recommendations by MOCHA system type

Format of recommendations*MOCHA System type							
			MOCHA system type				Total
			Combined	GP-lead	Pediatrician-lead	Other	
Format of recommendations[a]	Scientific format	Count	11	11	8	2	32
		% within system type	31,4%	33,3%	25,8%	33,3%	
	Administrative format	Count	12	6	9	2	29
		% within system type	34,3%	18,2%	29,0%	33,3%	
	Popular format	Count	10	10	9	1	30
		% within system type	28,6%	30,3%	29,0%	16,7%	
	Personal format	Count	2	6	5	1	14
		% within system type	5,7%	18,2%	16,1%	16,7%	
Total		Count	35	33	31	6	105

Percentages and totals are based on responses.

a. Group
